# Combined single-cell quantitation of host and SIV genes and proteins *ex vivo* reveals host-pathogen interactions in individual cells

**DOI:** 10.1371/journal.ppat.1006445

**Published:** 2017-06-27

**Authors:** Diane L. Bolton, Kathleen McGinnis, Greg Finak, Pratip Chattopadhyay, Raphael Gottardo, Mario Roederer

**Affiliations:** 1 US Military HIV Research Program, Henry M. Jackson Foundation, Walter Reed Army Institute of Research, Silver Spring, Maryland, United States of America; 2 ImmunoTechnology Section, Vaccine Research Center, NIAID, NIH, Bethesda, Maryland, United States of America; 3 Fred Hutchinson Cancer Research Center, Seattle, Washington, United States of America; Duke University Medical Center, UNITED STATES

## Abstract

CD4 T cells harboring HIV-1/SIV represent a formidable hurdle to eradicating infection, and yet their detailed phenotype remains unknown. Here we integrate two single-cell technologies, flow cytometry and highly multiplexed quantitative RT-PCR, to characterize SIV-infected CD4 T cells directly *ex vivo*. Within individual cells, we correlate the cellular phenotype, in terms of host protein and RNA expression, with stages of the viral life cycle defined by combinatorial expression of viral RNAs. Spliced RNA^+^ infected cells display multiple memory and activation phenotypes, indicating virus production by diverse CD4 T cell subsets. In most (but not all) cells, progressive infection accompanies post-transcriptional downregulation of CD4 protein, while surface MHC class I is largely retained. Interferon-stimulated genes were also commonly upregulated. Thus, we demonstrate that combined quantitation of transcriptional and post-transcriptional regulation at the single-cell level informs *in vivo* mechanisms of viral replication and immune evasion.

## Author summary

HIV-1, and its simian counterpart, SIV, infect and kill CD4 T cells, resulting in their massive depletion that ultimately leads to AIDS in the absence of antiretroviral therapy. With effective therapy, these cells are largely preserved, but a subset harbors latent virus that can persist for decades and reemerge upon therapy interruption, preventing HIV-1 cure. To prevent or eliminate productive cellular infection, there is tremendous demand to identify host factors expressed by these cells *in vivo*, which may serve as unique biomarkers or drug targets. Here we provide the first detailed combined transcriptomic and protein expression profile of SIV-infected cells directly *ex vivo* using novel single-cell technologies. Our survey of activation markers, interferon-stimulated genes, and viral restriction factors identified multiple host genes differentially expressed by SIV-infected cells and will inform future therapeutic strategies.

## Introduction

CD4 T lymphocytes that support HIV-1/SIV replication are central to the development of AIDS-defining illness as well as to the establishment of cell-associated viral reservoirs that persist despite years of antiretroviral therapy [1, 2]. Despite the clinical importance of these infected T cells, their properties are poorly defined at the cellular level due to the difficulty of characterizing them *in vivo* or directly *ex vivo* [3]. Barriers include their low frequency, estimated at 10^−3^–10^−6^ during chronic untreated HIV-1 infection [2, 4, 5], and lack of defining markers on their surface. Consequently, most data about infected cells is derived from either *in vitro* infection models or analysis of *ex vivo* bulk cell populations comprised mostly of uninfected cells.

*Ex vivo* studies employing methodology to specifically identify and characterize rare *in vivo* HIV-1/SIV-infected cells, as defined by expression of viral RNA, DNA or protein, are essential to gaining a better understanding of cells harboring virus. To date, only a few studies have accomplished this feat, and typically only a small number of surface markers were measured. From these, there is compelling evidence for cell surface CD4 (and CD3 for SIV) downregulation, a hallmark of *in vitro* HIV-1/SIV infection, *in vivo* [6–10], although an earlier report demonstrated CD4 retention [4]. MHC class I downregulation, another well-described *in vitro* phenomenon, has also been observed *ex vivo*, albeit subtly and not consistently in all hosts [7, 8]. Markers of T cell exhaustion (CTLA-4, PD-1, and TIGIT), peripheral follicular helper cells, Th17 cells [10], T cell memory, and activation (HLA-DR) also appear elevated. These basic phenotyping findings warrant more extensive investigation examining a greater number of markers and including the application of more sensitive methodology not reliant on viral protein detection to identify infected cells.

To further overcome this long-standing challenge to the field and establish a more detailed profile of elusive *in vivo* infected cells, we integrated two complementary approaches into a single technology, measuring the simultaneous expression of surface proteins (by flow cytometry) and over 90 host genes (by highly multiplexed qPCR) with single-cell resolution [11]. Using PCR assays specific for multiple forms of viral RNA, we identify SIV-infected cells directly *ex vivo* in different stages of the viral life cycle spanning early to highly productive states. Cell surface protein and transcriptional profile is compared across each infection stage to determine differential expression patterns associated with infection in individual cells. Moreover, we demonstrate post-transcriptional regulatory events in single infected host cells and correlate these events with viral gene expression.

## Results

### Quantitative multiplex SIV and host RNA expression within single CD4 T cells directly *ex vivo*

Progression through the HIV/SIV life cycle is characterized by sequential accumulation of multiply-spliced, singly-spliced, and unspliced viral RNA (vRNA), which thereby distinguish discrete infection stages. We used RT-qPCR assays to identify cells transcribing SIV by expression of spliced (*tat/rev*, *env)*, unspliced (*gag*), and total (*LTR*) vRNA (**S1 Fig**). *In vitro*, cell-associated spliced viral transcript expression followed expected kinetics during SIV_mac239_ infection of rhesus macaque PBMCs (**S1 Fig**) [12, 13], and reverse transcriptase inhibition blocked de novo *tat/rev* expression, confirming specificity for transcription from proviral DNA. In sum, the spliced vRNA assays identify active viral transcription, while *gag* and *LTR* detect more prevalent vRNA species not necessarily specific for gene expression.

**Fig 1:**
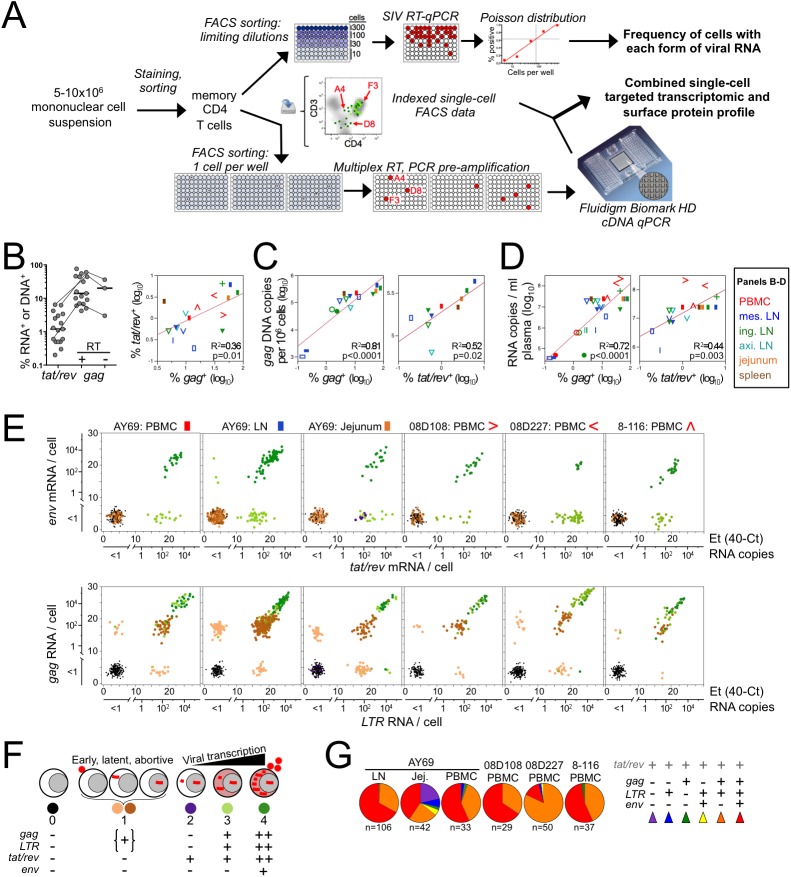
*in vivo* SIV-infected cell frequency and single-cell viral RNA expression. SIV-infected macaque specimens from 14 animals were surveyed for spliced and unspliced nucleic acid positive memory (CD95+) CD4 T-cells by limiting dilution FACS sorting and qPCR. Animals are described in S1 Table. **(A)** Schematic of the experimental workflow. **(B)** The frequency of *tat/rev+* and *gag+* cells are plotted alongside (left) and against each other (right), against total proviral DNA (*gag*) in bulk sorted memory CD4 T-cells **(C)**, and against concurrent viremia **(D)**. *gag* DNA^+^ cell frequency, determined by excluding reverse transcriptase (“RT–“), is also shown for a subset in (B); bars represent means. In bivariate plots, each animal is represented by a unique symbol and chronic infection by circles. **(E)** Single-cell SIV transcript levels within memory CD4 T cells from d10 SIVmac251 (AY69, n = 3 tissues) and d14 SIVsmE660 (PBMC, n = 3 animals) infected rhesus macaques (subset from B-D). The relative (Et) and absolute quantity of *env* and *tat/rev* (top) and *gag* and *LTR* (bottom) transcripts per cell is shown. Undetectable mRNA is plotted as a scatter near the origin for visualization. Symbol color corresponds to the number and type of viral transcripts detected in each cell; number of cells analyzed is provided in S3 Table. **(F)** Pictorial of viral life cycle with corresponding combination of viral genes present and symbol colors used herein. See also S1 Fig. **(G)** Pie charts depict the proportion of *tat/rev*^*+*^ cells expressing additional viral genes. Experiments were performed once for each animal and all data is shown.

We determined the frequency of infected cells *in vivo* during acute and chronic SIV infection of 14 rhesus macaques (*Macaca mulatta*; **S1 Table**), which reproduce most clinical and virological features of HIV-1 infection in humans [14]. Unmanipulated viable memory CD4 T cells from multiple tissues were sorted by flow cytometry at serial 3-fold dilutions in replicate to estimate the percent positive for spliced or unspliced vRNA (**Fig 1A**, top). *tat/rev* (multiply-spliced) RNA^+^ cells ranged from <0.01 to 6.4% (mean 2.0%) of memory CD4 T cells at 9-14d post-infection (**Fig 1B, S2 Fig**). On average, *gag*^*+*^ cells were present at ~10-fold higher frequency than *tat/rev*^+^ cells, comprising 0.2–80% (mean 26%) of memory CD4 T cells. Control experiments performed in the absence of RT for three lymph node samples yielded a two-fold reduction in *gag*^*+*^ cells (**Fig 1B;** 3%, 20%, and 36%), similar to but slightly less than previously reported frequencies of viral DNA^+^ T cells during acute SIV infection [15]. We attribute the DNA signal in our assay primarily to cytoplasmic reverse transcription products rather than integrated provirus as the latter is not efficiently recovered by the cell lysis protocol (**S3 Fig**), which may explain our lower DNA values. Not surprisingly, the frequencies of *tat/rev*^+^ and *gag*^*+*^ cells were strongly correlated with one another as well as with both total cell-associated proviral DNA measured in bulk memory CD4 T-cells and plasma viremia (**Fig 1C and 1D**). These results are consistent with virus production by *tat/rev*^+^ cells detected by our assay and recapitulate similar correlations observed in HIV-1 infection [16, 17].

To characterize viral gene expression in individual cells, we measured co-expression of vRNAs in FACS-sorted single cells directly *ex vivo* from six acutely SIV-infected macaque specimens (**Fig 1A, bottom**). Notably, this quantitation is sensitive and linear at the single-copy per cell level [11]. PBMC, lymph node (LN), and jejunum tissues were chosen for analysis based on predetermined infected cell frequencies ≥1% within memory CD4 T cells (**Fig 1B**). Four distinct subsets of vRNA+ cells were apparent based on the quantity and identity of vRNA species within a cell: 1) *gag*^*+*^ and/or *LTR*^*+*^, spliced vRNA^–^; 2) *tat/rev*^*+*^ only; 3) *tat/rev*^+^*env*^−^ and *gag*^*+*^ and/or *LTR*^*+*^; and 4) *tat/rev*^+^*env*^+^ and *gag*^*+*^ and/or *LTR*^*+*^ (**Fig 1E and 1F**). The profile of cells positive for *gag*, *LTR*, or both, in the absence of either of the spliced transcripts (hereafter referred to as stage 1) is consistent with early, abortive, or latent infection [18, 19]. In cells positive for multiple vRNAs, vRNA levels were highly correlated with one another (**S4 Fig**). 10^2^−10^5^ vRNA copies were expressed per cell, similar to estimates from HIV-infected cells [17]. Compared to *tat/rev*^*−*^(stage 1) infected cells, *tat/rev*^*+*^ cells (stages 3–4) contained ~100-fold more *gag* RNA, indicating expression of large quantities of unspliced vRNA. Notably, the *env*^*−*^subset of *tat/rev*^*+*^ cells (stage 3) expressed less *tat/rev* per cell than the *env*^+^ (stage 4) cells, as would be expected early in the viral life cycle prior to nuclear export of partially processed vRNA and Tat-mediated transcriptional activation of the viral promoter. Thus the combination and quantitative expression of viral transcripts can be used to determine the stage of the viral life cycle in individual cells (**Fig 1F**).

Single-cell viral gene co-expression analysis among *tat/rev*^*+*^ cells revealed that the majority (40–70%) of *tat/rev*^*+*^ cells also expressed *gag*, *LTR*, and *env*, while the remainder was largely *env*^*–*^*gag*^+^*LTR*^*+*^ (25–45%) (**Fig 1G)**. An unusually low proportion of *env*^*+*^ cells (16%) was present in animal 08D227 (PBMC), despite abundant *tat/rev* RNA (**Fig 1E and 1G**). This may reflect viral sequence divergence from consensus SIVsmE660 in this animal, limiting detection by the *env* assay. Jejunum contained a unique subset (stage 2; 21%) in which *tat/rev* was the only vRNA detected, and at very low copies per cell. Together, these data further support *tat/rev* expression as a marker of virus-producing cells [18, 19], and we therefore consider these cells productively infected.

**Fig 2:**
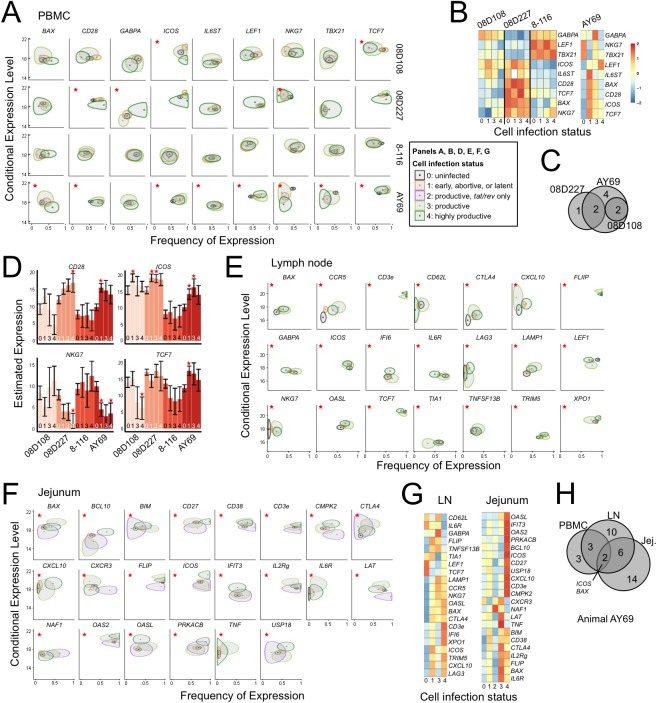
Differential host gene expression by *in vivo* SIV-infected T cells. **(A)** Genes showing a change in expression level or proportion in single cells from one or more infection states compared to uninfected cells in PBMC from one or more animals (n = 4). Significant changes (FDR < 0.1) are indicated (*). Plots display the MAST estimates of average frequency of expression (x-axis) and average expression level in cells when a gene is expressed (y-axis) for each gene by cell infection state (ellipse). Boundaries reflect the 90% bivariate Wald test confidence interval (Chi-square, 2 degrees of freedom). Contours with minimal or no overlap are more likely to be associated with a significant difference. The number of cells analyzed from each infection state is shown in S3 Table. (**B**) PBMC Z-statistics (standardized model coefficients) show expression across animals and infection stages by combining (using Stouffer’s method) the frequency of expression and continuous expression. Expression is mean centered by row. (**C**) Venn diagram of DE genes identified by the hurdle likelihood ratio test, common to PBMC from multiple animals. (**D**) Estimated average expression of four significant genes from (A) by cellular infection state. For each animal, significant differences from uninfected cells (at the 10% level after multiple testing adjustment across 92 genes tested) are marked with an asterisk. Unadjusted 95% confidence bounds are shown for the expression level. (**E-G**) Analyses of animal AY69 lymph node and jejunum as in (A-C). (**H**) Venn diagram of DE genes common across tissues within animal AY69. Two genes common to all tissues are indicated. Experiments were performed once for each specimen and all data is shown.

To identify cellular factors associated with viral infection at the single-cell level, differential host gene expression between uninfected cells and cells at each infection stage (**Fig 1F**) was assessed. Genes involved in T-cell activation, cell cycle regulation, signaling, viral restriction, and interferon response were selected for analysis (**S2 Table**). Differential gene expression was performed as previously described [20], with cell infection status modeled as a discrete covariate and each infection stage coefficient tested against uninfected cells (stage 0). Both the proportion of cells positive for a gene and the RNA copies per positive cell were considered. Among PBMC specimens, nine of the measured genes were altered in one or more infected cell populations, of which *CD28*, *ICOS*, *NKG7*, and *TCF7* differed in multiple animals (**Fig 2A–2D, S5 Fig**). Over 35 genes were differentially expressed by infected cells in lymph node and jejunum from animal AY69, and several of these genes were common to both tissues (**Fig 2E–2H**). As with PBMC, these included several activation markers and interferon-stimulated genes. Among productively infected (stages 3–4) cells, *BAX*, *CD28*, *CTLA4*, *FLIP*, *ICOS*, *CXCL10*, and *OASL* were upregulated. *BAX* and *ICOS* were differentially expressed in all three tissues form this animal. Genes encoding host proteins essential for viral infection and replication, the co-receptor *CCR5* and vRNA nuclear export factor *XPO1*, were also upregulated in lymph node infected cells. The largest magnitude differences between uninfected and productively infected cells were observed in the jejunum, with >2-fold increases in *BAX*, *CTLA4*, *ICOS*, *IFIT3*, *IL2RG*, *IL6R*, *LAT*, *OAS2*, *OASL*, *PRKACB*, *TNF*, and *USP18*. The considerable heterogeneity in host gene expression across animals, tissue type, and cell infection status indicates that viral expression occurs in a wide range of distinct subsets of CD4 T cells–making selective targeting of infected cells, necessary for cure modalities, a much greater hurdle.

### Single-cell post-transcriptional CD4 downregulation and MHC class I protein expression by SIV RNA^+^ CD4 T cells

**Fig 3:**
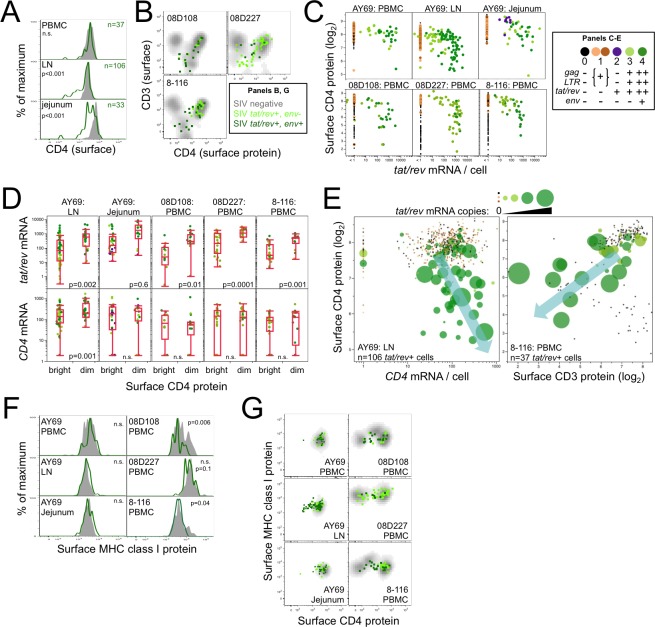
Post-transcriptional CD4 downregulation and MHC class I preservation on *in vivo* infected T cells. **(A)** FACS staining distribution of surface CD4 protein on SIV *tat/rev*^+^ productively infected (green; n = number of cells analyzed) memory CD95^+^ CD4 T cells from animal AY69 PBMC, lymph node, and jejunum compared to uninfected cells (gray). **(B)** FACS surface staining for CD3 and CD4 on *tat/rev*^*+*^ cells from memory CD3^+/-^ CD4 T cells in PBMC from 3 additional animals. **(C)** FACS CD4 surface fluorescence is plotted against *tat/rev* mRNA copies. Dot colors correspond to Fig 1F. **(D)** *tat/rev* (top) and *CD4* (bottom) mRNA copies per *tat/rev*^+^ cell is plotted by surface CD4 protein expression. **(E)** Surface CD4 protein (fluorescence) is plotted against *CD4* mRNA (left) or surface CD3 protein (right). Dot size corresponds to *tat/rev* mRNA copies per cell. FACS surface staining of surface MHC class I **(F)** and co-expression with surface CD4 **(G)** on *tat/rev*^*+*^ cells. Experiments were performed once for each specimen and all data is shown.

Combining flow cytometric single-cell immunophenotyping and RNA quantitation for each cell allows us to define post-transcriptional gene regulatory events within single cells. In *in vitro* models, HIV/SIV downregulate expression of several surface proteins on infected cells via putative post-translational mechanisms [21–29], but the degree to which this occurs *in vivo* is largely unknown. By comparing surface CD4 protein levels on uninfected and stage 3–4 infected CD4 T cells, we confirmed CD4 protein downmodulation on *in vivo* infected cells, but only on a subset of cells in jejunum and lymph node (**Fig 3A**). Indeed, the majority of *tat/rev*^+^ cells in PBMC (>95%) and lymph node (>70%) retained surface CD4 at levels comparable to uninfected cells. Expression was not reduced in stage 1 (spliced vRNA^-^) infected cells (**S6 Fig**). Because SIV Nef also downregulates CD3 [22], sorting from the three additional PBMC specimens included CD3-negative cells (**S2 Fig**) and surface CD4 was indeed diminished on 40–55% of stage 3–4 cells and downregulation correlated with decreased CD3 (**Fig 3B, S6 Fig**). Overall, the decrease in surface CD4 on downmodulated cells was ~90%, indicating residual surface CD4 despite active SIV transcription. Remarkably, the nine stage 2 (*tat/rev*^*+*^ only) cells observed only in jejunum expressed significantly more surface CD4 (and CD3) than uninfected cells, supporting the classification of this population as a unique subset of infected cells distinct from stages 3–4. Taken together, CD4 downmodulation *in vivo* is heterogeneous, varying across anatomical sites and among infected cells within a specimen, and indicates that this process may not be critical to viral pathogenesis.

To explore the mechanism of CD4 downmodulation within single cells infected *in vivo*, we quantified *tat/rev* and *CD4* transcript levels. At the one-cell level, surface CD4 protein was inversely associated with *tat/rev* RNA (**Fig 3C and 3D**). CD4 protein downmodulation was not due to decreased *CD4* mRNA (**Fig 3D and 3E, S6 Fig**); in fact, we observed an association with higher *CD4* mRNA levels. Moreover, we observed a progression of CD4 (and CD3) protein downmodulation, and increased *CD4* gene expression, in concert with viral transcription. These findings demonstrate CD4 regulation by SIV *in vivo* via either post-transcriptional or post-translational mechanisms. Of note, despite similar *tat/rev* expression (>1000 copies/cell) observed in AY69 PBMC, lymph node, and jejunum, surface CD4 was unchanged on *tat/rev*^+^ PBMC. Thus while high levels of tat/rev correlate with lower expression of CD4 protein, robust *tat/rev* expression is not sufficient for decreased expression, and therefore may require tissue-specific factors.

Post-translational downregulation of MHC class I HLA-A/B/C proteins from the surface of *in vitro* HIV/SIV-infected lymphocytes suggests that this may be a mechanism for evading cytotoxic T cell recognition *in vivo* [30–32]. Surprisingly, we found no consistent evidence of MHC class I downregulation on productively infected *tat/rev*^+^ cells, with the vast majority positive for HLA-A/B/C surface staining, even in cells that substantially downregulated CD4 protein (**Fig 3F and 3G, S6 Fig**). Decreased MHC class I surface expression was observed in *tat/rev*^+^ cells in two animals (08D108, p = 0.006 and 8–116, p = 0.04), although the amount of protein expressed per cell remained within the range observed for uninfected cells. Sequence analysis of the *nef* coding region in the SIVsmE660 inoculum did not reveal evidence of mutations known to impair MHC downmodulatory activity (**S6 Fig**) [23, 30]. Taken together, we find that MHC class I downregulation is limited *in vivo* and thus this proposed mechanism of immune evasion may not operate in pathogenic SIV infection.

### Surface activation and memory markers on SIV RNA^+^ CD4 T cells

**Fig 4:**
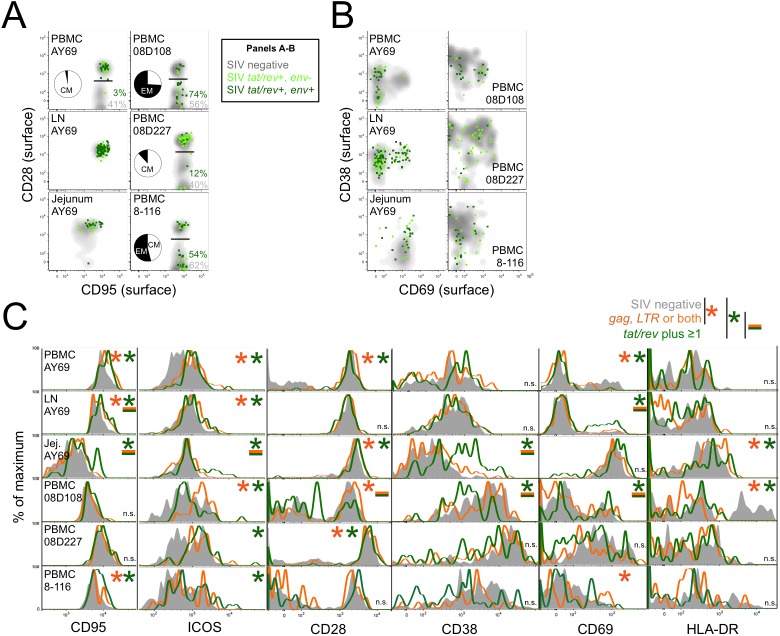
Surface phenotype of *in vivo* SIV-infected CD4 T cells. **(A)** Memory differentiation status of *tat/rev*^+^ infected cells. CD28 and CD95 surface protein expression by *tat/rev*^+^ cells (green) is overlaid atop the profile of SIV negative cells (gray). The percentage of cells negative for CD28 is indicated for each population. Inset pie charts depict the proportion of *tat/rev*^+^ cells that are central (CM; white) and effector memory (EM; black). See also S4 Table. **(B)** Surface staining for activation markers, CD38 and CD69, is shown for *tat/rev*^+^ cells. **(C)** Surface protein expression distribution of CD95, ICOS, CD38, CD69, and HLA-DR by uninfected (gray), *gag*^*+*^ and/or *LTR*^*+*^ (orange; early, latent or abortive infection), and *tat/rev*^*+*^ (green) cells. Significant differences were determined by ANOVA across the three populations (p<0.05) followed by Student’s t-test between populations (p<0.05, *). Experiments were performed once for each specimen (n = 6) and all data is shown.

To define phenotypic traits that distinguish infected cells, we measured expression of activation and differentiation surface markers. Productively infected *tat/rev*^+^ cells were nearly exclusively CD28^+^ central memory (CM) in lymph node and jejunum (**Fig 4A, S4 Table**). Among PBMC, 3–74% of *tat/rev*^+^ cells were effector memory (EM), suggesting preferential infection of CM, EM or no bias across hosts. The activation state of *tat/rev*^+^ cells was also diverse. In AY69, *tat/rev*^+^ jejunal cells largely expressed CD69 with variable CD38, while *tat/rev*^+^ PBMCs were exclusively CD69^–^, and lymph node was mixed (**Fig 4B**). *tat/rev*^+^ PBMCs from the other animals were also remarkably heterogeneous. Surprisingly, the presence of CD69^–^CD38^–^ *tat/rev*^+^ cells in multiple animals and tissues suggests that cellular activation may not be required for abundant viral gene expression *in vivo*. In addition, the activation profiles of uninfected T cells varied markedly among animals, likely reflecting variable degrees of host immune activation during acute infection. These findings are summarized in **S4 Table**.

These differential surface protein expression profiles were further quantified by comparing the staining distribution of uninfected, *tat/rev*^−^ (stage 1), and *tat/rev*^+^ (stage 3–4) infected cells. CD95 and ICOS showed higher expression on both *tat/rev*^+^ and *tat/rev*^−^ infected cells compared to uninfected cells (**Fig 4C**). Surface expression of CD38, CD69, and HLA-DR was also elevated on *tat/rev*^+^ cells in one or two specimens, while there was either no significant difference or diminished expression on *tat/rev*^+^ cells for other specimens. Of note, within animal AY69, specific activation markers were upregulated dependent on the tissue: CD69 in lymph node versus CD38 in jejunum. Overall, the broad distribution of these activation markers on the surface of *tat/rev*^+^ cells indicates highly variable expression among virus-producing cells. In most cases, differentiation and activation marker expression did not differ between productive *tat/rev*^+^ and non-productive *tat/rev*^−^ infected cells. Exceptions included elevated CD95, ICOS, and CD38 (jejunum) or CD95 and CD69 (lymph node) by *tat/rev*^+^ cells. Conversely, the stage 1 infected cells, which presumably represent cells at the time of or shortly after infection, exhibited significantly higher surface CD4 and CD3 relative to uninfected (and *tat/rev*^*+*^) cells in some specimens (**S6 Fig**). The distinct protein phenotype of stage 1 cells relative to uninfected cells with respect to CD4, CD3, CD95, and ICOS (among others shown in **Fig 4C**) provides independent validation of the *gag* and *LTR* assays to identify a discrete subset of infected cells.

## Discussion

We integrated single-cell transcriptomic and flow cytometric technologies and successfully quantified and characterized rare *in vivo* SIV-infected CD4 T cells, including demonstration of post-transcriptional gene regulation at the one-cell level. Within single cells, different combinations of viral RNA molecules were co-expressed in distinct patterns and quantities, consistent with a continuum of virus replication stages. Productively infected *tat/rev*^+^ cells commonly, but not universally, expressed elevated levels of host cell genes and proteins associated with T cell activation. Protein profiling revealed broad similarities to uninfected cells and, surprisingly, remarkable heterogeneity among *tat/rev*^+^ cells with respect to CD4 downregulation, memory phenotype, and activation state, along with a lack of consistent evidence of MHC class I downregulation, even within CD4^dim^ cells. Ample viral gene expression in memory cells devoid of multiple T cell activation surface markers indicates that an activated state is not required for productive CCR5-tropic virus infection, corroborating several previous studies primarily investigating CXCR4-tropic virus [33–36].

Our finding that MHC class I downregulation was limited on the surface of productively *in vivo* infected CD4 T cells is at odds with several prior studies demonstrating the importance of this SIV Nef function *in vivo* [30, 37]. In these studies, strong selective pressure resulted in either reversion of a Nef point mutation or compensatory mutations elsewhere in Nef that restored MHC-downregulating activity. However, it is also possible that other unknown Nef activities were affected by these mutations and contributed to the selective pressure. One limitation of our approach was the use of a pan-MHC class I antibody. Maintenance of nonclassical MHC class I molecules (e.g. *Mamu-E*) on the cell surface may mask downregulation of classical class I molecules and thus corroboration with additional antibody specificities is warranted. Nonetheless, our data calls into question the extent to which MHC class I is downregulated *in vivo* and the relevance of the hypothesis that the virus modulates the expression of MHC as a mechanism of immune escape.

Paradoxically, several ISGs with known antiviral properties were upregulated in productively infected cells, suggesting ineffective inhibition of SIV replication within CD4 T cells by these ISGs. Increased ISG expression may reflect a more activated state that predisposes cells to express viral genes. Alternatively, productive infection may directly or indirectly trigger ISG expression following autocrine or paracrine interferon signaling, respectively. It should be emphasized that gene expression differences between infected and uninfected cells may indicate pre-infection profiles, post-infection virus-induced modulation, or a combination thereof.

The phenotype and host/viral transcriptome of infected cells varied considerably across the different tissues analyzed. First, a rare subset of *tat/rev*^*+*^ infected cells lacking other vRNA molecules (stage 2) was present only in the jejunum, and not in PBMC or LN (**Fig 1**). This may reflect greater basal T cell activation in jejunum fostering low-level viral transcription without progression to a more productive state. Indeed, CD69 expression was greatest in jejunum, both among vRNA^−^ and vRNA^+^ cells (**Fig 4B**). Second, the largest fold-changes in host gene expression by infected cells were observed in the jejunum; specifically among cells in the highly productive stage 4 (**Fig 2G**). Third, some differentially expressed genes were upregulated among infected cells in one tissue, while downregulated in a different tissue (e.g. *IL6R*, *LEF1*, *NKG7*, and *TCF7*), even within the same animal. Fourth, CD4 downmodulation, while observed in all three tissues, was greater in jejunum than LN and PBMC. These inter-tissue differences do not appear to be associated with viral transcription levels as infected cells in each tissue expressed comparable quantities of the vRNAs measured. Rather, other factors inherent to the anatomical site, such as cellular activation, metabolism, cytokine milieu, and interferon-responsiveness may contribute to the divergent, tissue-specific properties of *in vivo* infected cells [38]. Given that the lymph node and jejunum analyses were derived from a single animal, these observations will need to be confirmed in additional animals in future studies.

Some caveats to our findings should be considered. First, the SIV gene expression assays do not distinguish between viral DNA and RNA. As a result, the *gag* and *LTR* assays, neither of which relies on RNA splicing, measure the presence of RNA, DNA, or both. Therefore positivity for these viral genes in the absence of spliced RNA (stage 1) is unlikely to reflect viral transcription and we refer these cells as most likely residing in early, latent, or abortive infection states. We cannot distinguish between these different infection stages using the current technology. For example, virion-derived genomic RNA, nascent cytoplasmic reverse transcribed proviral DNA, and silent integrated proviral DNA (although inefficiently detected in our system) would all be similarly characterized as *gag* and/or *LTR* positive. This distinction also has important implications for measuring infected cell frequencies. By capturing both genomic viral RNA and DNA, our approach may report higher values than previous studies reliant on DNA alone, particularly during acute viremia when virion RNA is pervasive. The reduction from 60% to 36% infection among memory CD4 T cells in one lymph node sample when vRNA was excluded is consistent with a substantial vRNA contribution.

Second, the viral life cycle staging we assigned to single infected cells should be considered a theoretical framework rather than a definitive consecutive series. Productive infection (stages 3 and 4), for example, may be followed by transition to a quiescent state in which low levels of unspliced RNA but little to no spliced RNAs are expressed (stage 1); a status that has been described in studies of treatment suppressed HIV-1 infection [18, 39]. However, given the context of untreated acute infection from which all of our single-cell analyses were derived, we believe the ordered staging likely applies to most cells. Viral cytopathic effects and cell-mediated immunity are expected to rapidly eliminate most stage 3–4 infected memory CD4 T cells.

Third, the number of host factors and cell types investigated in our single-cell approach was limited. Flow cytometric indexed single-cell sorting is currently limited to ~15 parameters, while the Fluidigm Biomark qPCR gene expression platform measures 96 user-determined genes. Furthermore, in the present study, for pragmatic reasons (to enrich for vRNA^+^ cells), we examined memory CD4 T cells at peak viremia time points. The extent to which our findings apply to naïve T cells, non-lymphoid cells, and post-acute infection settings requires further investigation.

Given the remarkable phenotypic and transcriptional diversity of *in vivo* infected cells observed here, therapeutic strategies that aim to target infected cells must address an eclectic mix of CD4 T cells. Future studies employing single-cell RNA-Seq technology and 35-40-parameter flow cytometry sorting will further increase the power of this approach, as will the incorporation of DNA assays to specifically identify latent infection. These data illustrate the increased information content from integrating these single-cell technologies for revealing immunobiology of viral infections, including, for the first time, the ability to quantify post-transcriptional regulation at the single-cell level. This technology will have broad applicability in defining regulatory normal and aberrant mechanisms in cell biology and pathogenic infections.

## Materials and methods

### In vitro SIV infection

Rhesus macaque PBMC were stimulated with PHA-P (Sigma L9017) for three days followed by spinoculation with SIVmac239 virus generated by plasmid transfection of 293T cells (ATCC CRL-3216). Infected cell cultures were maintained in recombinant human IL-2 (1 U/μl; R&D Systems 202-IL) and indinavir (2 μm) to limit virus replication to a single round. Lamivudine (3TC; 10μM) was used to prevent productive infection. Cells were lysed for RNA extraction at serial time points (RNaqueous kit, Ambion) or stained for intracellular Gag expression (BD Fix/Perm kit, Coulter KC57RD1 anti-p24). cDNA was synthesized from RNA using the DyNAmo cDNA Synthesis Kit (Thermo Scientific) as per the manufacturer’s instructions. Reverse transcription was primed with random hexamers. The following reagents were obtained through the NIH AIDS Research and Reference Reagent Program, Division of AIDS, NIAID, NIH: Indinavir Sulfate, Lamivudine.

### Quantitative RT-PCR for SIV gene expression

Viral genomic sequences from thirteen SIVmac251 and SIVsmE660 isolates were used to identify conserved sequences within individual SIV *tat/rev*, *env*, and *LTR* for maximal cross-reactivity. Probes for spliced genes span exon-exon junctions to minimize amplification of unspliced cDNA. Primer/probe sequences were optimized on bulk RNA extracts from SIVmac239 and SIVsmE660 *in vitro* infected cells. qPCR was performed using Platinum Taq polymerase as per manufacturer’s instructions (Life Technologies). Unspliced viral genomic RNA was measured using SIV *gag* qPCR primer and probe sequences previously described [40]. The SIV *LTR*.*U3* qPCR primer probe set consisted of the following: forward: equimolar amounts of 5’-TAC CCA GAA GAG TTT GGA AGC AAG TCA-3’ and 5’-TAC CCA GAA GAG TTT GGT AGT AAG TCA-3’; reverse: equimolar amounts of 5’-TTG TCA GCC ATG TTA AGA AGG CCT CTT G-3’ and 5’-TTG TCA GCC ATT TTA WWA AKG CCT CTT G-3’; probe: equimolar amounts of 5’-CTG TCA GAG GAA GAG GTT AGA AGA AGG CTA AC-3’ and 5’-TTG TCA GAG GAA GAG GTA AAG AGA AGG CTA AC-3’. The SIV *env* assay consisted of the following: forward: 5’-AGA GGC CTC CGG TTG CA-3’; reverse: equimolar amounts of 5’-CTT ACT TGT TTG ATG CAG AAG ATG-3’ and 5’-CTT ACT TGT TTG ATG CAG RAR RTG-3’; probe: 5’-TTA GYC TTA GYC TTY TTC GGA GTT CTT CTT-3’. The SIV *tat/rev* assay: forward: equimolar amounts of 5’-GAA CTC CGA AAA AGG CTA AGG CTA ATA CA-3’ and 5’-GAA CTC CGA ARA AGR CTA AGR CTA ATM CA-3’; reverse: equimolar amounts of 5’-CCK TCT CCT TCT TCT CCT TCT TTG GTT-3’ and 5’-CCG TCT CRT TCT TTG CCT TCT CTG GTT-3’; probe: equimolar amounts of 5’-CTGCATCAAACAACCCATATCCAACAGGACC-3’ and 5’-CTG CAT CAA ACA A ATC CCT ATC CAC AAG GRC C-3’.

### Animals and SIV infection

Fourteen colony-bred Indian-origin male and female rhesus macaques (age 3 to 9 years old) were infected with various SIV strains administered as follows: intravenous SIV_mac251_ (100 MID_50_; VRC animal protocols 150.1, 356, 417), intrarectal SIV_mac251_ (AID_50_; VRC 211.3), and intrarectal SIV_smE660_ (AID_30_; VRC 332) (**S1 Table**). Virus preparations of 1.0 ml were inoculated via the saphenous vein or a lubricated feeding catheter inserted into the rectum. Infection duration ranged from 4 days to 8 months.

### Cell sorting and flow cytometry

Viable mononuclear cell suspensions were stained with fluorochrome-conjugated monoclonal antibodies from BD Biosicences (San Jose, CA, unless otherwise indicated) to CD4 (clone OKT4, BioLegend #317434), CD3 (SP34-2, #557757), CD8 (RPA-T8, in-house Qdot655 conjugate), CD28 (CD28.2, #555730), CD95 (DX2, #558814). Dead cells and monocytes were excluded by: LIVE/DEAD Aqua (ThermoFisher) and CD14 (M5E2 in-house Ax700PE). Memory (CD95^+^) CD8^-^ CD3+ T-cells were sorted on a BD FACSAria (BD Biosciences) as either serial limiting 3-fold dilutions ranging from 3–1000 cells per well for frequency calculations or as single cells (n = ~1850) for Biomark transcriptomic analysis in 96-well plates (**Fig 1A,** top and bottom, respectively). The total number of cells sorted for limiting dilution analysis is shown in **S1 Table** for each specimen. For example, a typical limiting dilution sort plate consisted of 6 replicates each of 500 and 150 cells/well and 12 replicates each of 50, 15, and 5 cells/well (n = 4740 cells analyzed). For animals 08D108, 08D227, and 8–116, CD3^-^ cells were included to account for CD3 downmodulation by SIV Nef [22] while CD16 (3G8, BioLegend #302048) and CD20 (2H7, in-house Ax700PE) were used to exclude NK and B cells. Additional markers recorded for indexed phenotyping of single cells mapped by well position were HLA-DR (L243, #339194), CD69 (FN50, in-house Ax594), ICOS (C398.4A, BioLegend #313505), CD38 (OKT10, NHP Reagent Resource, PE), CCR7 (150503, in-house Ax680), and MHC class I (W6/32, BioLegend #311430) [41]. All clones were previously determined to cross-react with rhesus macaques (NHP Reagent Resource) and lots were individually titrated to identify optimal concentration per test. Cells were deposited into 96-well PCR plates (GeneMate, Bioexpress) for immediate lysis (within ~100 μsec from protein detection) and RNA extraction. 3D8 cells containing a single copy of integrated SIV DNA [42] were FACS sorted in control experiments and as standard curves for absolute DNA quantitation. To minimize changes in cell RNA and protein expression prior to analysis, cell samples were maintained on ice at all times, with the exception of the surface stain (15 min) and elapsed sorting time (~30 min). All samples were previously cryopreserved, which was determined not to impact viral gene expression (**S1 Table**). 96-well PCR collection plates were maintained on pre-chilled aluminum blocks during and after the sort. FACS data was analyzed using FlowJo v9.8. Cells unlikely to be CD4 T-cells were excluded from downstream FACS analyses if they met the following three criteria: CD4 fluorescence intensity below cut-off (<175 for AY69 LN; <500 for 08D227, 08D108, 8–116), undetected *CD4* mRNA, and undetected *CD40LG* mRNA expression. Undetected *CD3E* mRNA was used as an additional exclusion criteria for animals 08D108, 08D227, and 8–116. *tat/rev*^*+*^ cells with downregulated CD4 (“dim”) were defined by fluorescence staining intensity as follows: <1100 in lymph node and PBMC; <3000 in jejunum.

### Limiting dilution and single cell qRT-PCR

RNA from sorted cells collected in 10 μl of SuperScript III-Platinum Taq One-step qRT-PCR mastermix (Life Technologies) was directly reverse transcribed and PCR pre-amplified, as previously described [11], with the following thermocycling conditions: 50^°^C for 15 minutes, 95^°^C for 2 minutes, followed by 18 cycles of 95^°^C for 15 seconds and 60^°^C for 4 minutes. Gene-specific primers were used for priming both the RT and PCR pre-amplification reactions. cDNA was diluted 5-fold and subjected to either conventional qPCR on an ABI 7900 real-time PCR instrument for 40 cycles or multiplexed qPCR on a Fluidigm Biomark HD system. The frequency of cells expressing a given viral transcript was calculated by plotting the fraction of replicate wells positive by RT-qPCR at each limiting dilution versus the number of cells sorted per well, followed by Poisson distribution analysis of the cell dilution corresponding to one positive cell per well, based on the expected frequency of 63.2% of wells positive at that dilution (**Fig 1A**). All qPCR was performed in dedicated plasmid-free workspaces. Multiple PCR experiments were negative for all viral genes, including animal AY69 pre-infection PBMC and day 4 post-infection AZ26 and ZC55 PBMC (**S1 Table**), indicating minimal SIV RNA or DNA PCR contamination.

Quantitative gene expression in single cells was measured using the Fluidigm Biomark microfluidic chip platform (**Fig** 1**A**). TaqMan assays (Life Technologies) consisted primarily of FAM-MGB probes that span exon-exon junctions (**S2 Table**) and passed qualification tests to establish both efficient and linear amplification as well as multiplexing capability [11]. Assays not specific for exon-exon junctions or otherwise capable of detecting genomic DNA (suffix “s1” and “g1”) were considered unlikely to influence our gene expression results because: 1) genomic DNA is not readily accessed by our cell lysis protocol (**S3 Fig**), and 2) all cells are expected to contain the same number of genomic DNA copies. Samples and assays were loaded on the 96.96 Biomark Dynamic Array Chip for Gene Expression following manufacturer’s instructions. Briefly, 3.6 ul of 1:5 diluted cDNA was mixed with 4.4 ul of a 1:10 mixture of Fluidigm Sample Loading Regent and Taq Universal PCR Master Mix to create the real-time reaction sample mix. Equal volume of 20X TaqMan assay and Fluidigm GE Assay Loading Reagent prior were combined to generate the 10X assay mix. 5 μl of each mix was loaded on the chip inlets. Biomark qPCR was performed using the GE 96.96 Standard V.1 protocol with 40 cycles of PCR and analyzed using the auto initialized Ct thresholds for each detector. Relative qPCR values are reported as expression threshold (Et, where Et = 40-Ct). Absolute RNA copies were calculated as 2^(Et-13)^, given Et = 13 corresponds to a single copy of RNA using this protocol [11]. Positivity for either *gag* or *LTR* within single cells likely reflects variable qPCR assay detection at ≤2 copies.

### Differential gene expression analysis

Single cell gene expression data was analyzed using the hurdle model framework implemented in the MAST package [43]. Data was filtered for cells with low cellular detection rate (CDR) (< 12.5% of genes expressed, 9 cells) [43]. Additional cells not flagged by this method that expressed a frequency of genes similar to that of excluded events were also omitted from analysis to eliminate all potential outlier cells (**S7 Fig**). For samples sorted to include CD3^-^ cells, there was substantial variation that could not be explained by potential confounders such as animal or chip after filtering. A subset of cells from stage 0 (viral RNA negative) from different animals formed a distinct cluster in tSNE space (**S7B Fig**, red, 55 cells) that was not associated with cellular detection rate. Because we are interested in differences between cellular infection states within each animal, we assessed gene expression differences between this outlier cluster and cells not in the cluster (**S7C Fig**). Monocyte genes, *NLRP3* and *NOD2*, were enriched in this cluster, while T cell genes, *CD3*, *CD28*, and *CD40LG*, were relatively absent, indicating that these cells are most likely not T cells. This provided biological justification for excluding this cell cluster from the downstream analysis. The number of cells in each infection state from each animal is shown in **S3 Table**. Differential gene expression analysis was performed as previously described [20, 43]. Gene expression in infected cell subsets, defined by the number and type (unspliced or spliced) of viral genes expressed (**Fig 1E and 1F**), was modeled using the hurdle generalized linear model implemented in MAST and differences between infected cell subsets in each animal and tissue were tested using the hurdle likelihood ratio test. The MAST hurdle model tests for a difference in proportion (percent cells positive for a gene) and a difference in conditional mean (Et) and provides a combined likelihood ratio test that includes both sources of information. Multiple testing adjustment was applied using the Benjamini Hochberg FDR method across all tests and significance was called at a false discovery rate threshold of 10%. Differentially expressed genes were visualized by plotting point estimates of group effects from the continuous and discrete parts of the model with simultaneous 90% bivariate confidence ellipses (Chi-square, 2 d.f.) around the estimates. These generally agree with the likelihood ratio test, but can be less conservative for small sample sizes in some instances. The estimated average expression level for cells from each subject and group was calculated from the hurdle model (controlling for CDR) fit to the single cell expression data, as described [43–45]. Briefly, the estimate combines the discrete and continuous estimates, with standard errors derived via the delta method, and can be interpreted as the average expression that would be expected to be observed in a bulk cell experiment.

### Statistics

Significant differences in gene or surface protein (mean fluorescence) expression between two cell populations were determined by the Student’s t-test (p<0.05). For protein comparisons involving three or more populations, differences were first assessed by ANOVA (p<0.05) followed by Student’s t-test. All single cell protein and gene expression experiments were performed on six biological replicates with multiple cells measured per animal. Sample size was determined by available resources, i.e. number of SIV-infected animals and viable cells from each specimen. Given previous experience analyzing Biomark single-cell data, the available number of cells was deemed sufficient.

### Ethics statement

All animals were cared for in accordance with guidelines set by the NIH Guide for the Care and Use of Animals. The NIH Vaccine Research Center IACUC approved all animal protocols and procedures. Animal protocols included ASPs 150, 356, 417, 211, and 332.

### Data availability

All data for the single-cell gene expression analysis are available at https://zenodo.org/record/803385.

## Supporting information

S1 Fig SIV gene assays and *in vitro* SIV gene expression detection by real-time qPCR. (**A**) qPCR assays to detect the indicated SIV RNA molecules are depicted by arrows positioned at location of forward (right arrow) and reverse (left arrow) primers for amplification of transcripts encoding the gene(s) in the corresponding color. For spliced transcripts *env* and *tat/rev*, probes span the splice junction. Shading indicates region amplified; introns are represented by dashed lines. Viral life cycle stages characterized by expression of each SIV RNA is shown at right. (**B**) SIV gene expression in rhesus macaque PBMC infected *in vitro* with SIVmac239 in the absence or presence of 3TC (open symbols). Bulk RNA harvested at the indicated time post-infection was reverse transcribed and analyzed for viral and *alb* cDNA by qPCR in triplicate; mean and standard deviation are plotted. Relative gene expression was calculated as: 2^(Et_vRNA_—Et_alb_). (**C**) *In vitro* SIV gene expression analysis as in (B) with SIV *env* assay included.
(TIF)

S2 Fig Flow cytometric gating scheme for isolation of memory CD4 T cells. (**A**) FACS gating tree employed for limiting dilution and single cell sorting of memory CD4 T cells from jejunum, lymph node, and PBMC from animal AY69. (**B**) FACS gating tree employed for limiting dilution and single cell sorting of memory CD4 T cells from PBMC of animals 08D108, 08D227, and 8–116.
(TIF)

S3 Fig Inefficient recovery of integrated SIV proviral DNA using standard one-step RT-PCR lysis protocol. 3D8 cells containing a single copy of integrated SIV DNA were FACS sorted at 30 cells per well (n = 6 replicates) followed by lysis for RNA / DNA recovery by the indicated protocol and qPCR for integrated SIV DNA using Alu-LTR nested PCR. Protocols included: 1) “Manufacturer’s lysis” (blue), ThermoFisher SuperScript III—Platinum Taq One-step qRT-PCR protocol as described in Materials and Methods; 2) “Proteinase K lysis” (green), commonly used for harvesting cell-associated DNA; and 3) “modified manufacturer’s lysis” (red), which incorporates a Proteinase K lysis step into the one-step qRT-PCR protocol. The relative gene copies is plotted as 2^(Et)^, where Et = 40-Ct. Alu-LTR copies increased 10,000-fold by addition of a Proteinase K nuclear membrane lysis step to the manufacturer’s lysis protocol. Low level qPCR amplifcation of unintegrated LTR sequences is known to occur in this assay via Alu-independent read-through transcription of RNA or DNA primed by a single LTR primer in the first round PCR, and subsequent qPCR amplification by the LTR-specific forward and reverse primers during the second round. This likely explains the signal in samples lysed with the manufacturer’s standard protocol, in which cytoplasmic viral RNA containing LTR would be readily accessible. All lysis conditions were subjected to the same number of pre-amplification PCR cycles and qPCR template was normalized by cellular input.
(TIF)

S4 Fig Correlation between viral genes co-expressed within a cell. Bivariate plots of the SIV RNA expression by individual memory CD4 T cells isolated from d10 SIVmac251-infected AY69 rhesus macaque lymph node. RNA copies expressed per cell is plotted for each viral gene versus all other viral genes. Linear regression analysis is shown in red with correlation coefficient and p-value indicated. Dot colors correspond to infection states depicted in Fig 1F.
(TIF)

S5 Fig Single cell differential host cell gene expression across vRNA+ cell subsets. Violin plots depict single-cell continuous and proportional gene expression for PBMC (**A**), AY69 lymph node (**B**), and AY69 jejunum (**C**). Each cell is represented by a dot and infection state is indicated along the x-axis. Blue lines and gray shading indicate empirical mean and 90% confidence intervals. Asterisk indicates FDR <10% in combined likelihood ratio test comparisons relative to uninfected cells (0).
(TIF)

S6 Fig CD4, CD3 downregulation on *in vivo* infected T cells. FACS staining distribution of surface CD4 **(A)** and CD3 **(B)** protein on memory CD8^-^ CD3^+^ T cells sorted from SIV-infected rhesus macaque specimens described in Fig 1E. The staining profile of cells positive for *gag* or *LTR* (orange), *gag* and *LTR* (brown), *tat/rev* only (purple), and *tat/rev* plus at least one additional SIV gene (green) is overlaid atop that of uninfected cells within the same sample (gray). The number (n) of RNA^+^ cells depicted is indicated. **(C)** Single-cell FACS CD4 surface staining is plotted against *CD4* mRNA copies for the samples in Fig 3A and 3B. **(D)** Surface MHC class I protein staining versus CD4 downregulation status. Histogram and dot plot coloring corresponds to Fig 1E and 1F. **(E)** SIVsmE660 stock sequence did not present any *nef* mutations known to alter MHC-I downregulation (red). Mutations known to alter CD4 and CD28 downregulation (blue) were also wild-type.
(TIF)

S7 Fig Single cell gene expression quality control. (**A**) Heat map depicts expression of 96 genes (columns) in gray scale for each PBMC cell (rows) analyzed on the Biomark for animal AY69. Cells flagged by the algorithm for expression of an unusually high or low number of genes are indicated at left in red, while cells with more typical expression profiles are indicated in blue. (**B**) For animals 08D108, 08D227, and 8–116, principal component analysis was used to identify additional outlier SIV RNA^-^ cells (red) not associated with cellular detection rate. (**C**) Violin plots of genes differentially expressed by the outlier cell cluster (“+”) in (B) compared to non-outlier cells (“-”).
(TIF)

S1 Table SIV-infected macaque specimens analyzed for frequency of cells expressing viral RNA. (TIF)

S2 Table Real-time qPCR TaqMan assays used for quantitative gene expression. (TIF)

S3 Table Number of single cells included in differential gene and protein expression analyses from each specimen. (TIF)

S4 Table Memory and activation phenotype of SIV-infected CD4 T cells by infection stage for each specimen analyzed at single-cell resolution. (TIF)
