# Supplementary figures and images for: Combined single-cell quantitation of host and SIV genes and proteins ex vivo reveals host-pathogen interactions in individual cells
Source: PLoS Pathog. 2017 Jun 27;13(6):e1006445. doi: 10.1371/journal.ppat.1006445 (PMC5507340; doi:10.1371/journal.ppat.1006445)

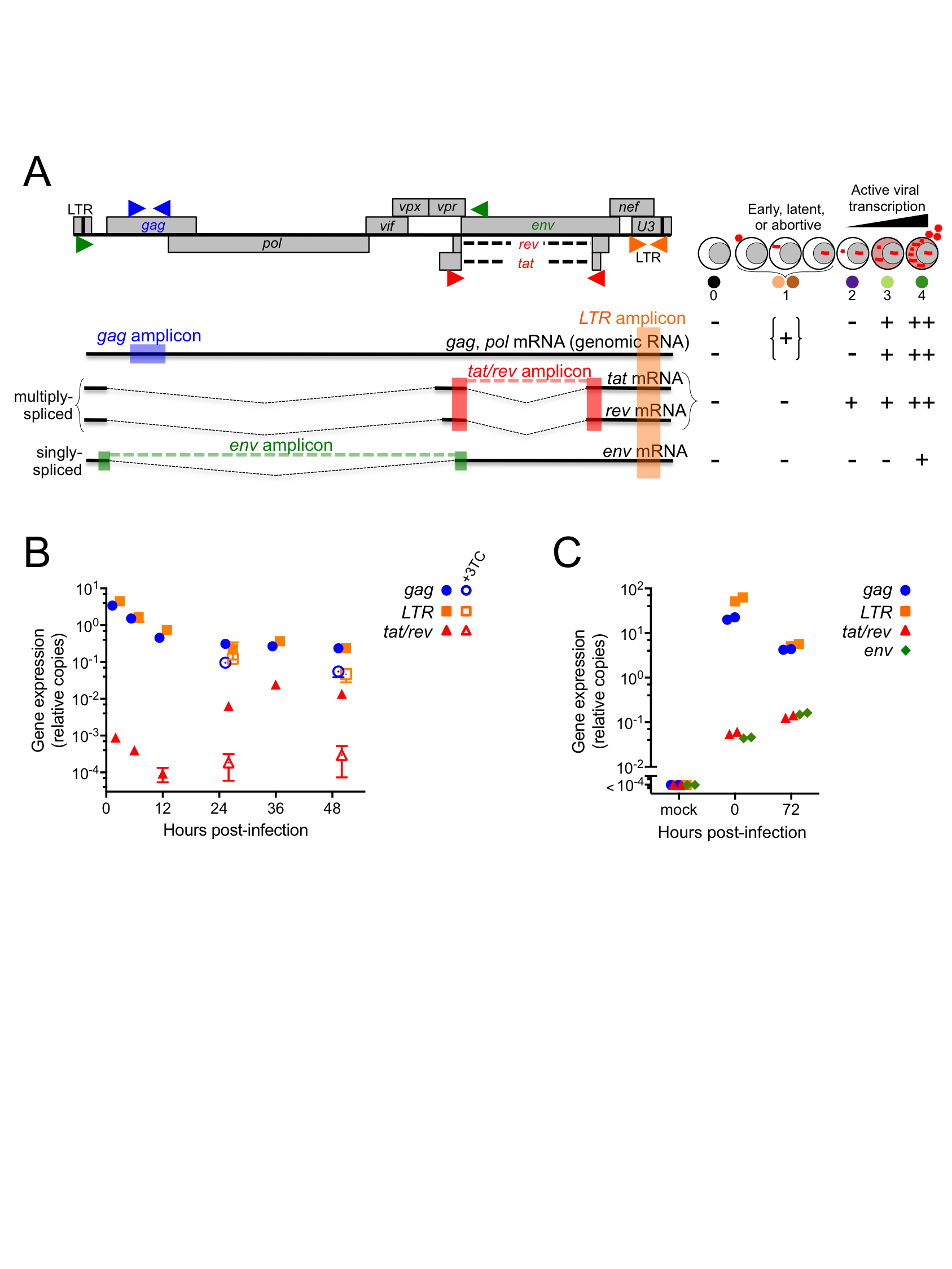

Supplement: S1 Fig — (A) qPCR assays to detect the indicated SIV RNA molecules are depicted by arrows positioned at location of forward (right arrow) and reverse (left arrow) primers for amplification of transcripts encoding the gene(s) in the corresponding color. For spliced transcripts env and tat/rev, probes span the splice junction. Shading indicates region amplified; introns are represented by dashed lines. Viral life cycle stages characterized by expression of each SIV RNA is shown at right. (B) SIV gene expression in rhesus macaque PBMC infected in vitro with SIVmac239 in the absence or presence of 3TC (open symbols). Bulk RNA harvested at the indicated time post-infection was reverse transcribed and analyzed for viral and alb cDNA by qPCR in triplicate; mean and standard deviation are plotted. Relative gene expression was calculated as: 2^(EtvRNA—Etalb). (C) In vitro SIV gene expression analysis as in (B) with SIV env assay included. (TIF) [file ppat.1006445.s001.tif]

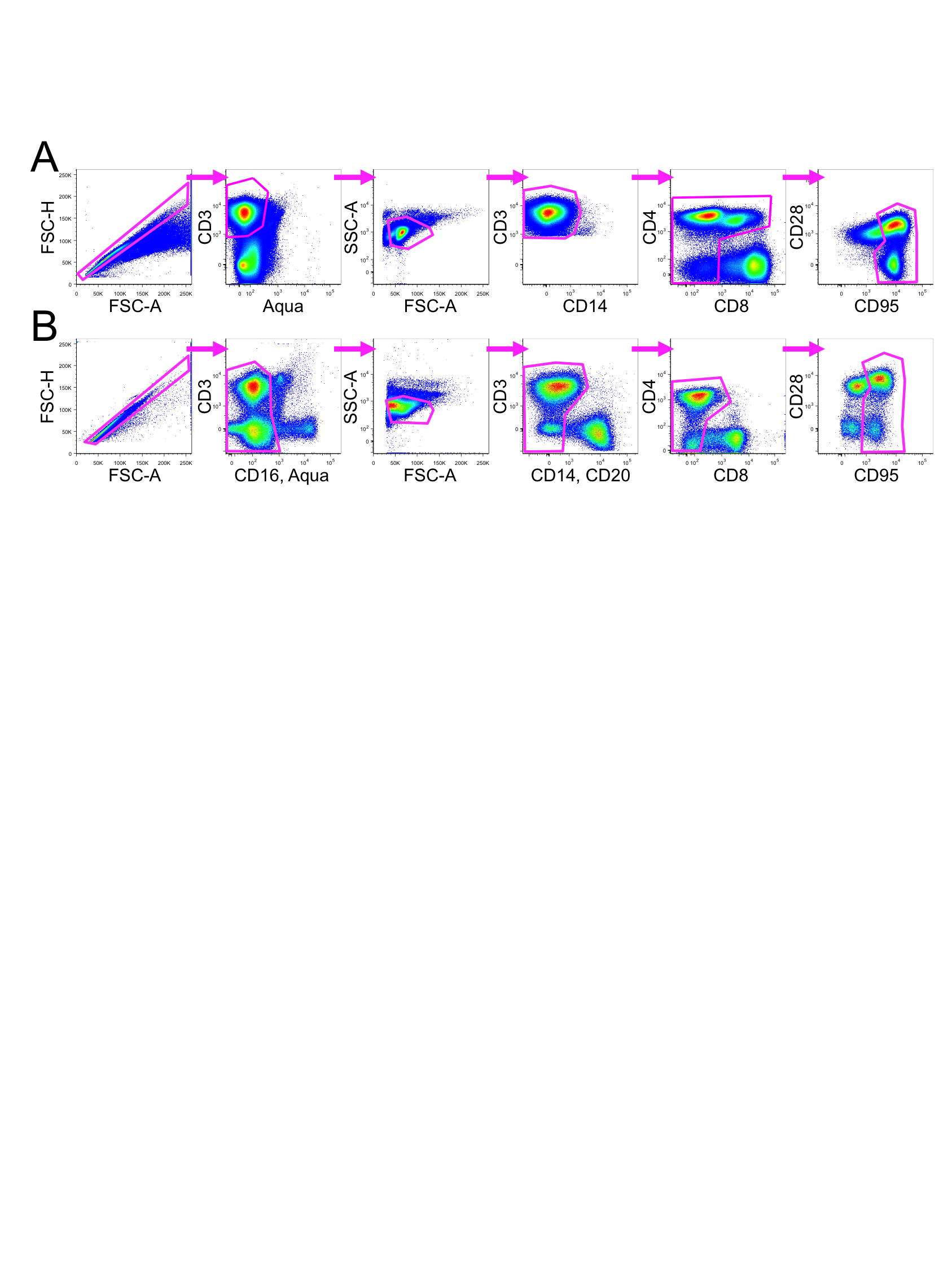

Supplement: S2 Fig — (A) FACS gating tree employed for limiting dilution and single cell sorting of memory CD4 T cells from jejunum, lymph node, and PBMC from animal AY69. (B) FACS gating tree employed for limiting dilution and single cell sorting of memory CD4 T cells from PBMC of animals 08D108, 08D227, and 8–116. (TIF) [file ppat.1006445.s002.tif]

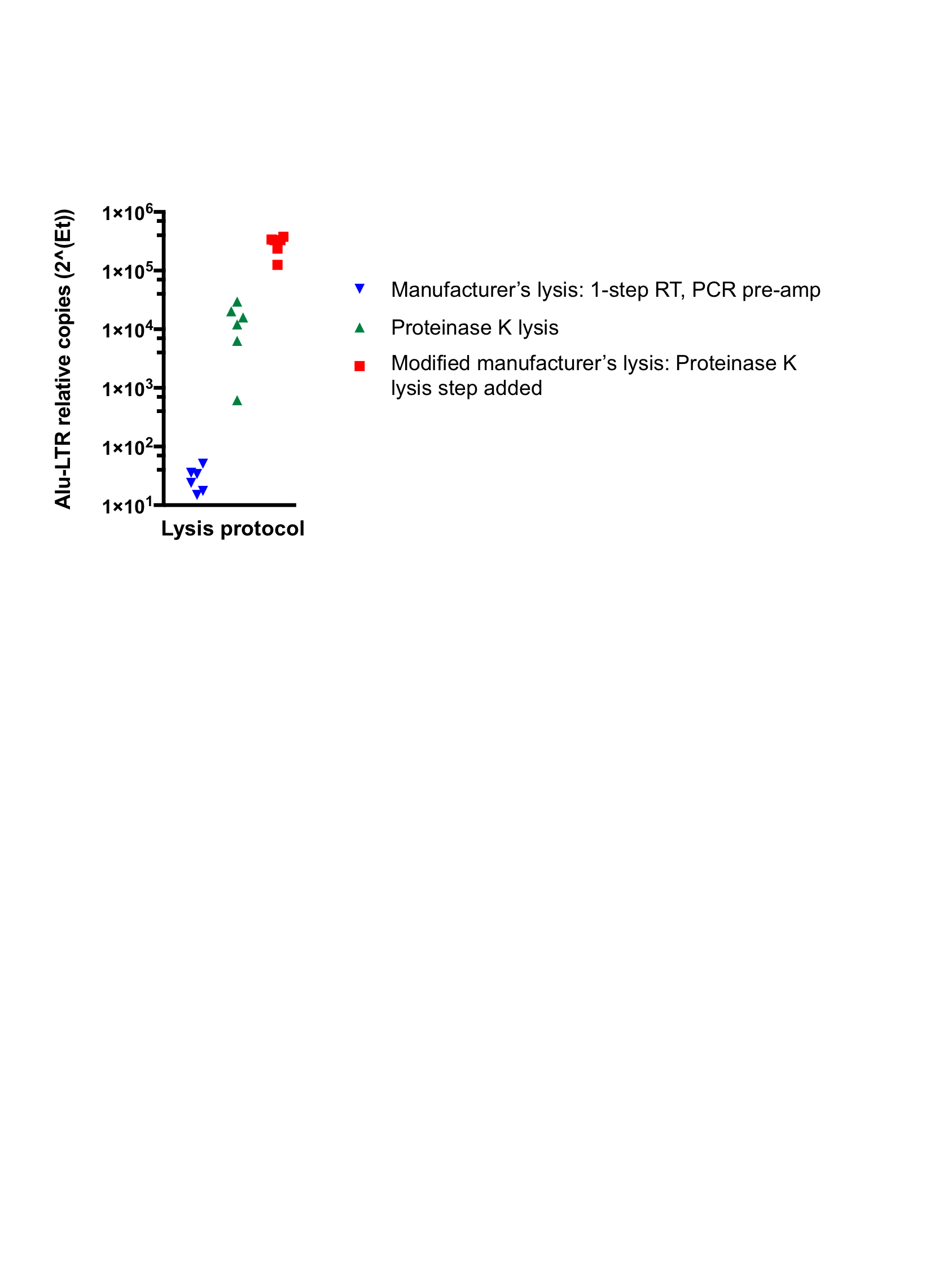

Supplement: S3 Fig — 3D8 cells containing a single copy of integrated SIV DNA were FACS sorted at 30 cells per well (n = 6 replicates) followed by lysis for RNA / DNA recovery by the indicated protocol and qPCR for integrated SIV DNA using Alu-LTR nested PCR. Protocols included: 1) “Manufacturer’s lysis” (blue), ThermoFisher SuperScript III—Platinum Taq One-step qRT-PCR protocol as described in Materials and Methods; 2) “Proteinase K lysis” (green), commonly used for harvesting cell-associated DNA; and 3) “modified manufacturer’s lysis” (red), which incorporates a Proteinase K lysis step into the one-step qRT-PCR protocol. The relative gene copies is plotted as 2(Et), where Et = 40-Ct. Alu-LTR copies increased 10,000-fold by addition of a Proteinase K nuclear membrane lysis step to the manufacturer’s lysis protocol. Low level qPCR amplifcation of unintegrated LTR sequences is known to occur in this assay via Alu-independent read-through transcription of RNA or DNA primed by a single LTR primer in the first round PCR, and subsequent qPCR amplification by the LTR-specific forward and reverse primers during the second round. This likely explains the signal in samples lysed with the manufacturer’s standard protocol, in which cytoplasmic viral RNA containing LTR would be readily accessible. All lysis conditions were subjected to the same number of pre-amplification PCR cycles and qPCR template was normalized by cellular input. (TIF) [file ppat.1006445.s003.tif]

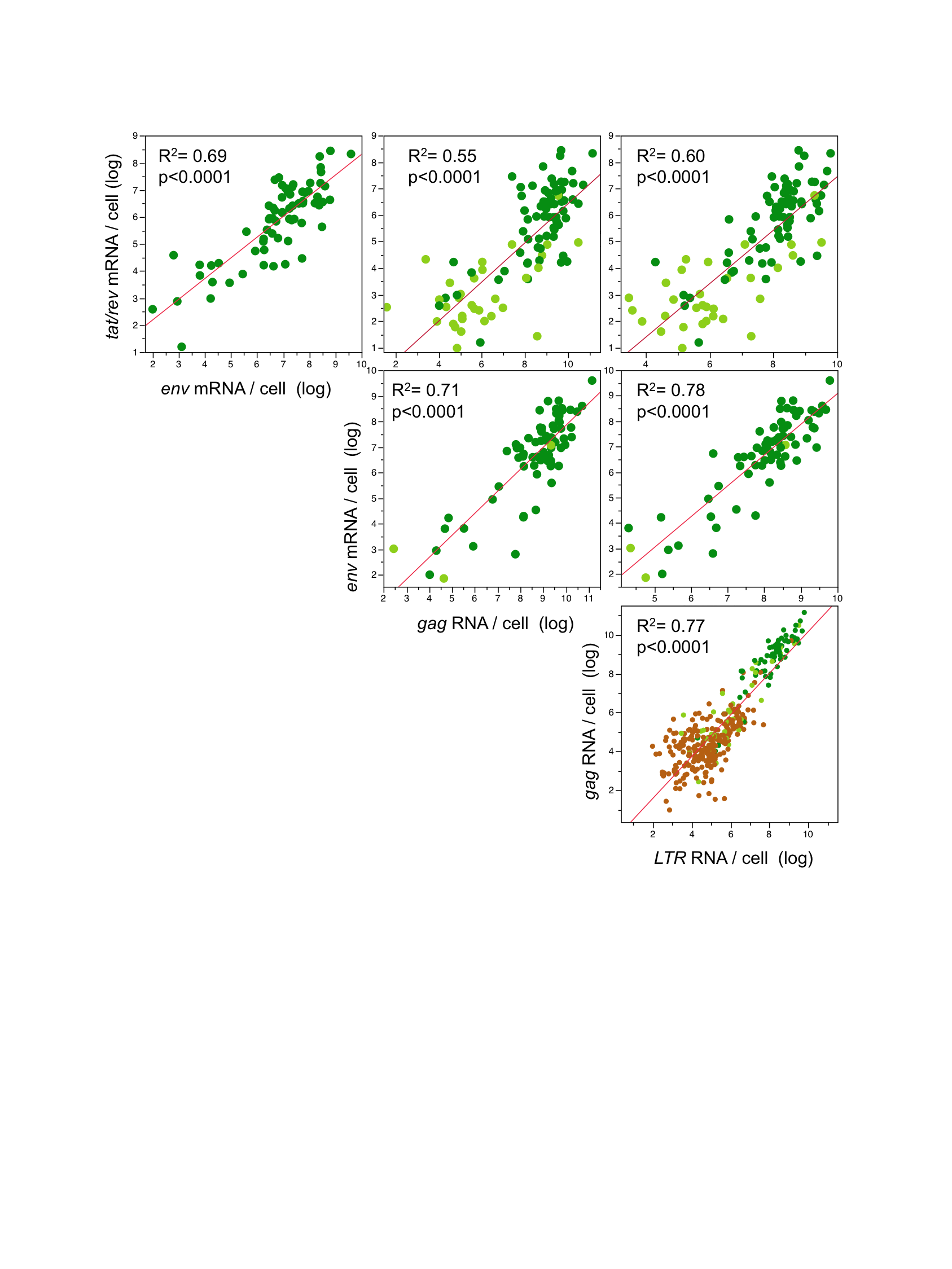

Supplement: S4 Fig — Bivariate plots of the SIV RNA expression by individual memory CD4 T cells isolated from d10 SIVmac251-infected AY69 rhesus macaque lymph node. RNA copies expressed per cell is plotted for each viral gene versus all other viral genes. Linear regression analysis is shown in red with correlation coefficient and p-value indicated. Dot colors correspond to infection states depicted in Fig 1F. (TIF) [file ppat.1006445.s004.tif]

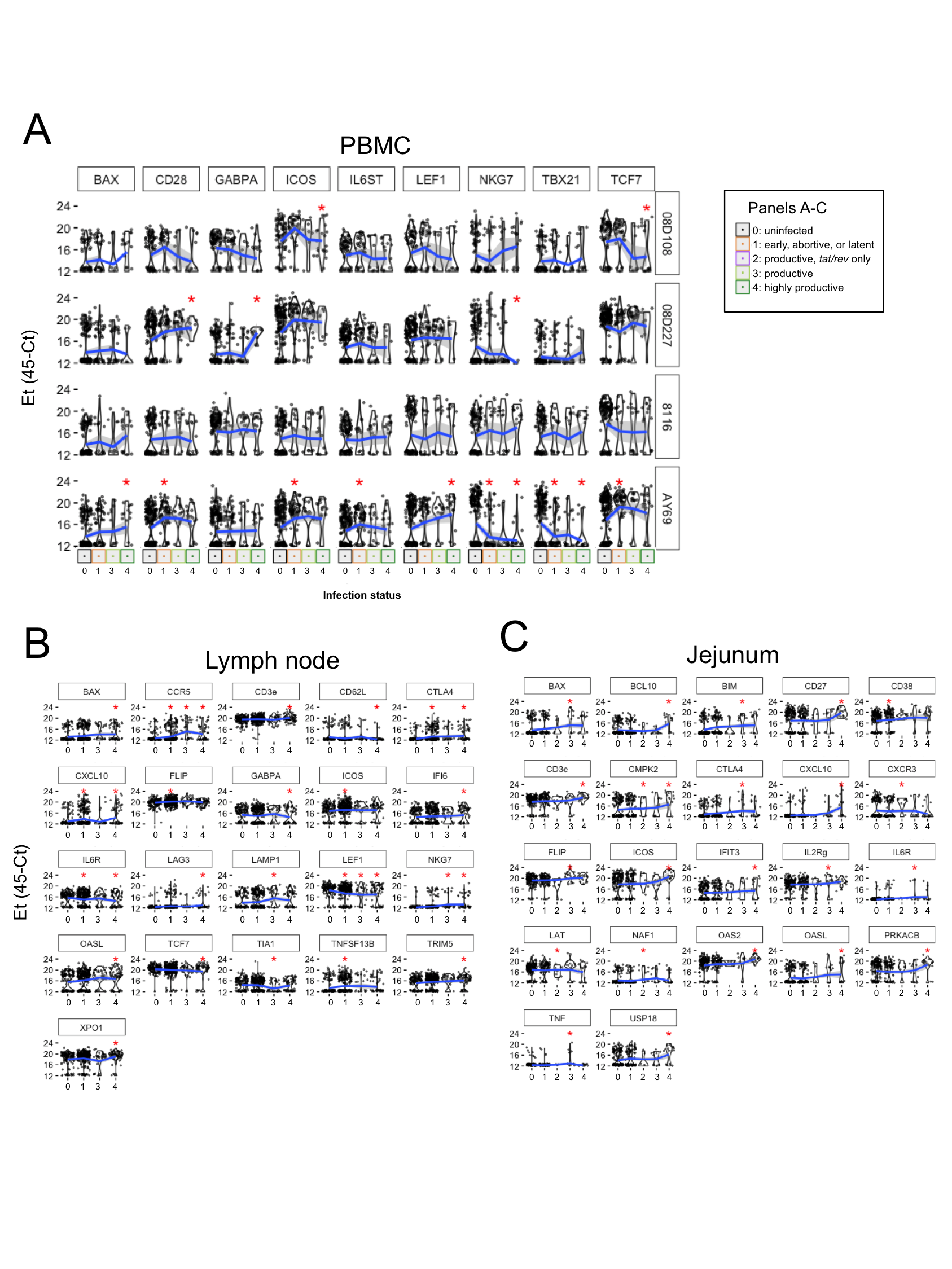

Supplement: S5 Fig — Violin plots depict single-cell continuous and proportional gene expression for PBMC (A), AY69 lymph node (B), and AY69 jejunum (C). Each cell is represented by a dot and infection state is indicated along the x-axis. Blue lines and gray shading indicate empirical mean and 90% confidence intervals. Asterisk indicates FDR <10% in combined likelihood ratio test comparisons relative to uninfected cells (0). (TIF) [file ppat.1006445.s005.tif]

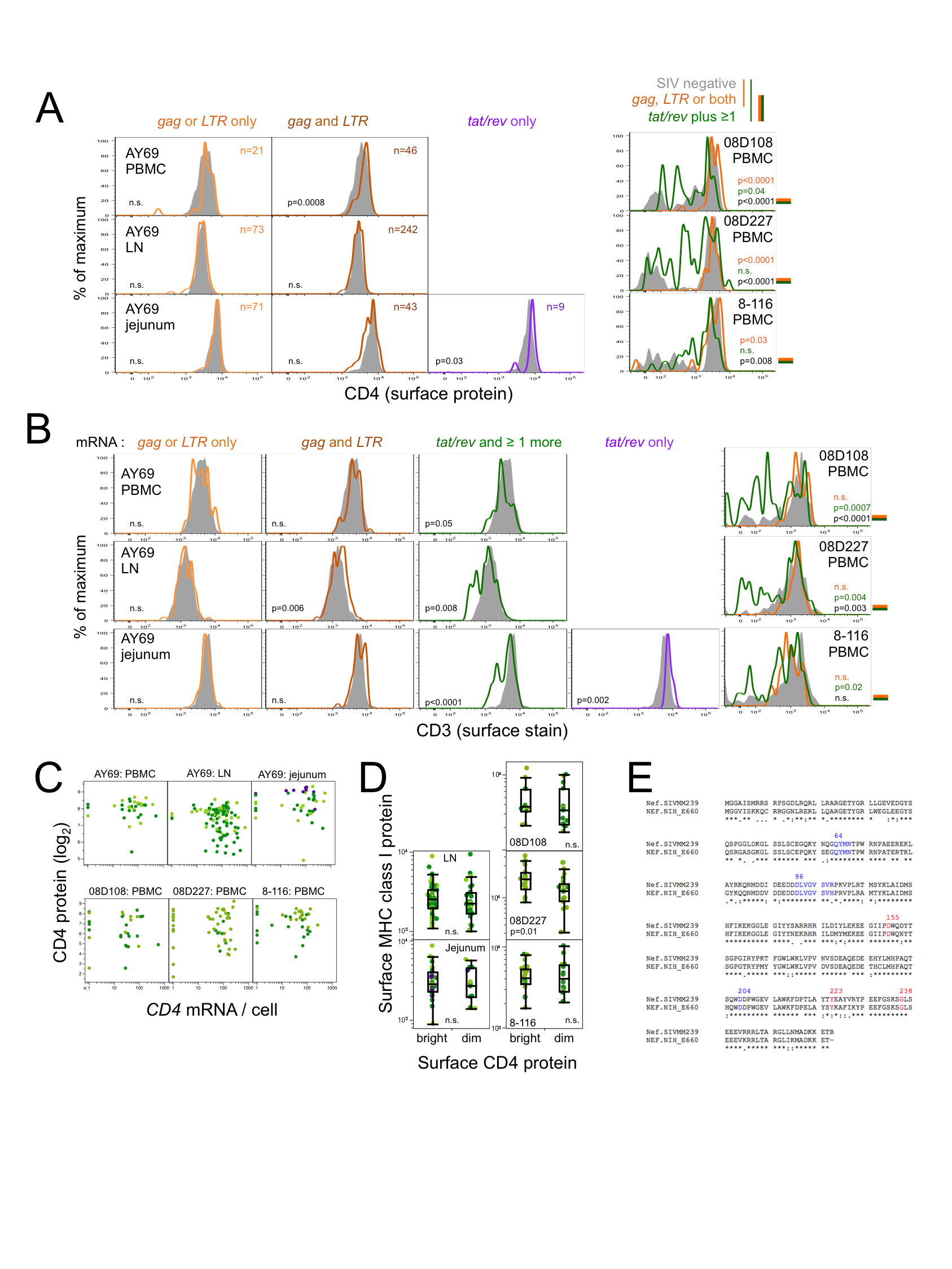

Supplement: S6 Fig — FACS staining distribution of surface CD4 (A) and CD3 (B) protein on memory CD8- CD3+ T cells sorted from SIV-infected rhesus macaque specimens described in Fig 1E. The staining profile of cells positive for gag or LTR (orange), gag and LTR (brown), tat/rev only (purple), and tat/rev plus at least one additional SIV gene (green) is overlaid atop that of uninfected cells within the same sample (gray). The number (n) of RNA+ cells depicted is indicated. (C) Single-cell FACS CD4 surface staining is plotted against CD4 mRNA copies for the samples in Fig 3A and 3B. (D) Surface MHC class I protein staining versus CD4 downregulation status. Histogram and dot plot coloring corresponds to Fig 1E and 1F. (E) SIVsmE660 stock sequence did not present any nef mutations known to alter MHC-I downregulation (red). Mutations known to alter CD4 and CD28 downregulation (blue) were also wild-type. (TIF) [file ppat.1006445.s006.tif]

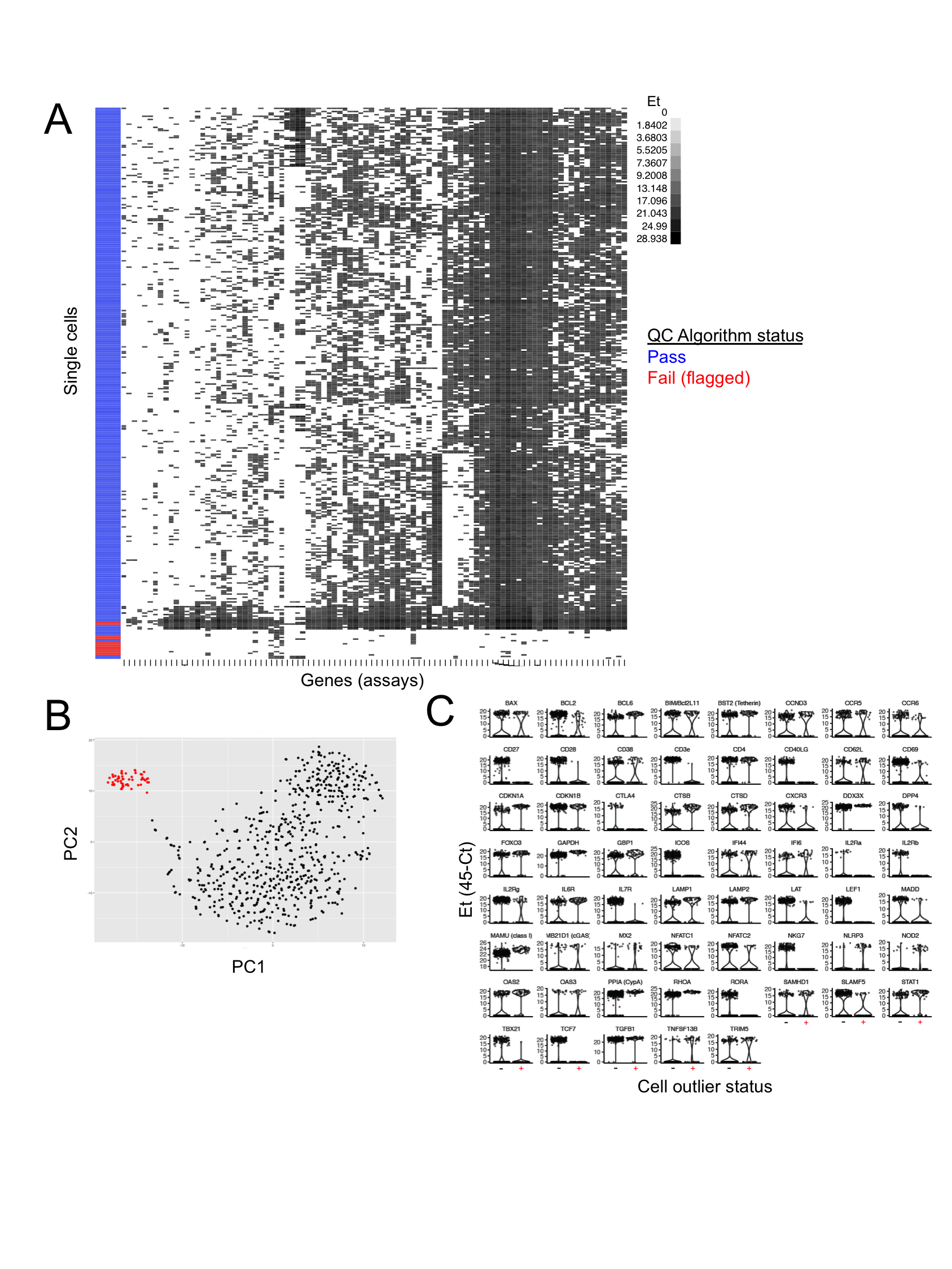

Supplement: S7 Fig — (A) Heat map depicts expression of 96 genes (columns) in gray scale for each PBMC cell (rows) analyzed on the Biomark for animal AY69. Cells flagged by the algorithm for expression of an unusually high or low number of genes are indicated at left in red, while cells with more typical expression profiles are indicated in blue. (B) For animals 08D108, 08D227, and 8–116, principal component analysis was used to identify additional outlier SIV RNA- cells (red) not associated with cellular detection rate. (C) Violin plots of genes differentially expressed by the outlier cell cluster (“+”) in (B) compared to non-outlier cells (“-”). (TIF) [file ppat.1006445.s007.tif]

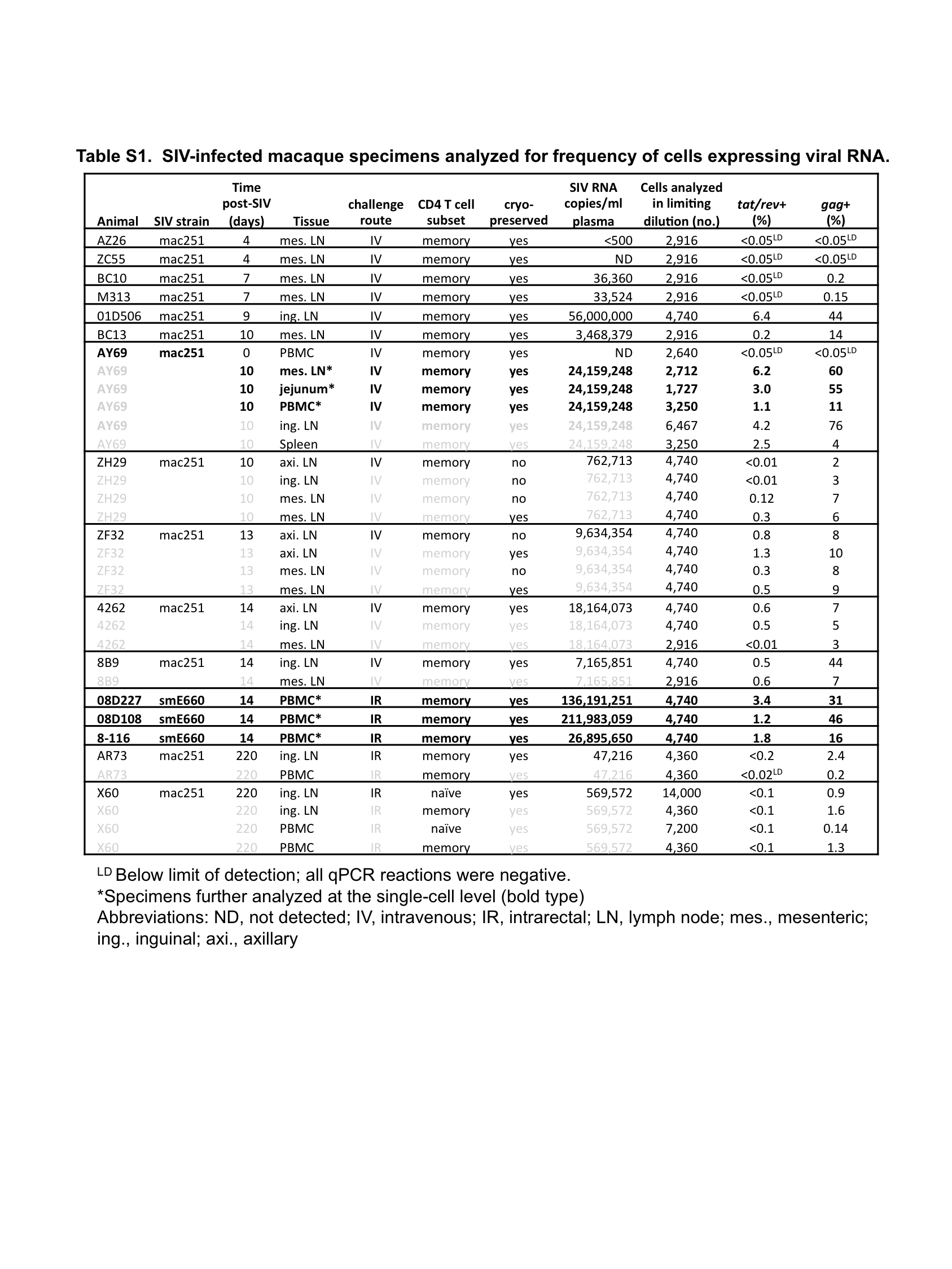

Supplement: S1 Table — (TIF) [file ppat.1006445.s008.tif]

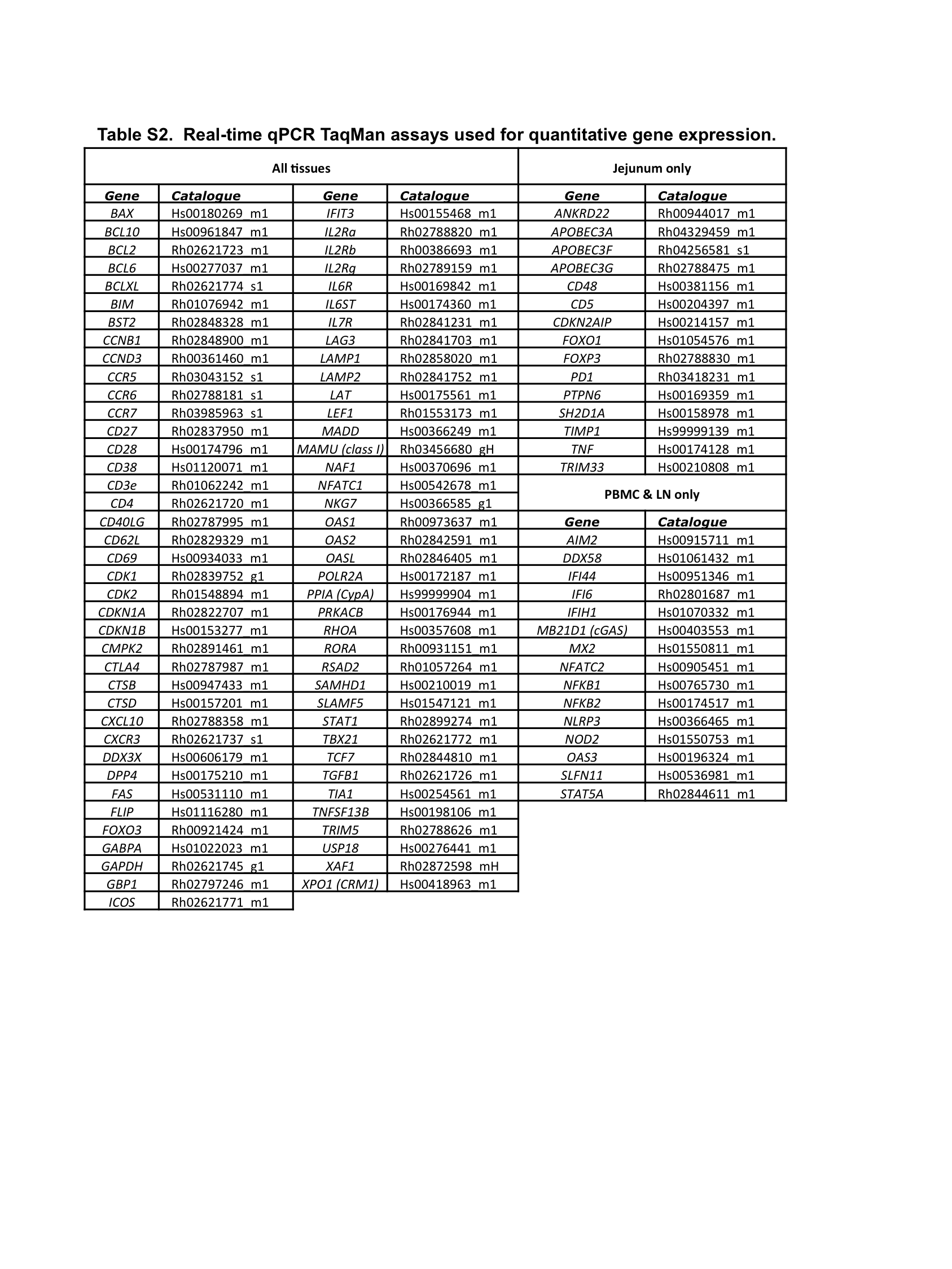

Supplement: S2 Table — (TIF) [file ppat.1006445.s009.tif]

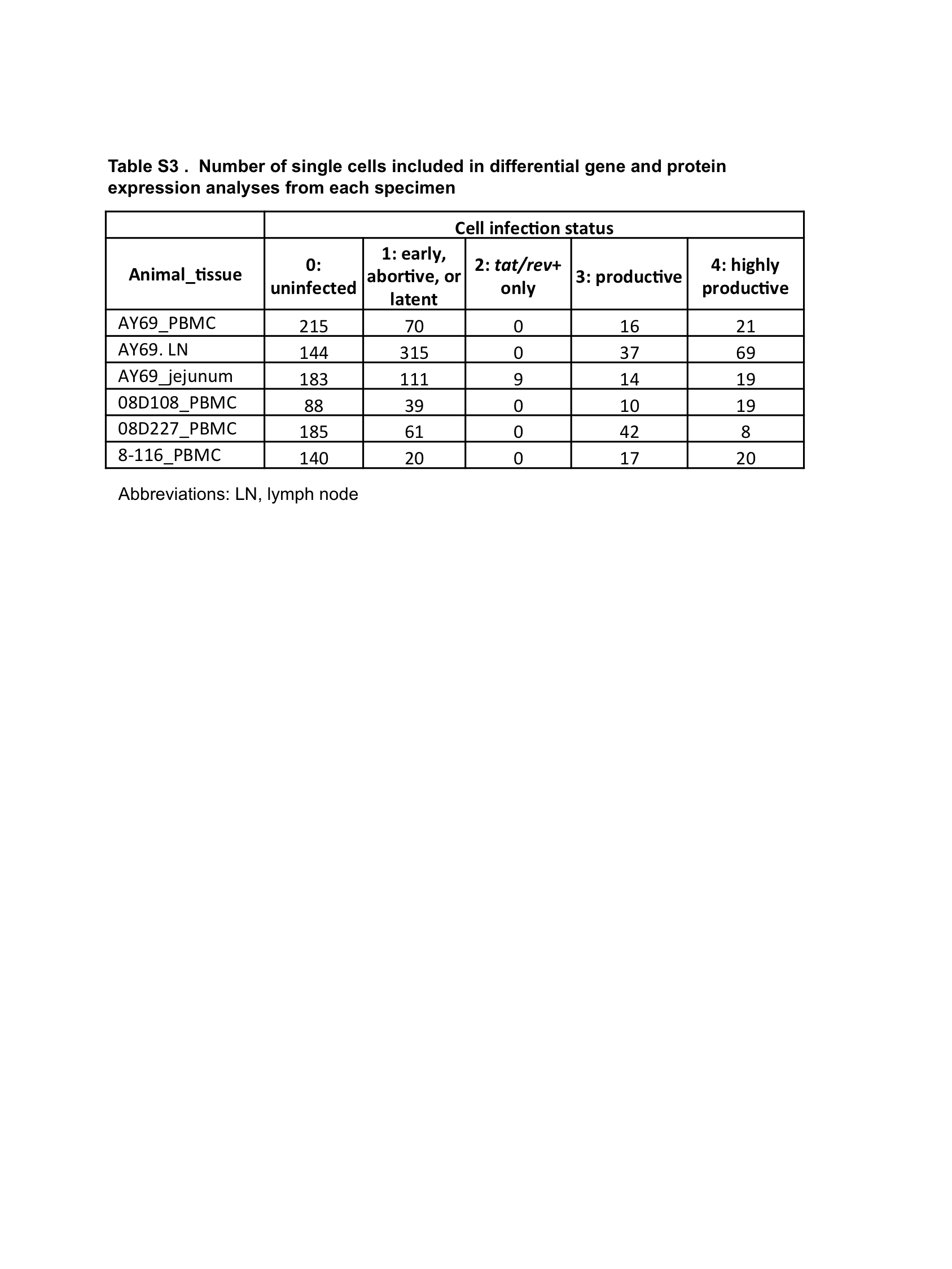

Supplement: S3 Table — (TIF) [file ppat.1006445.s010.tif]

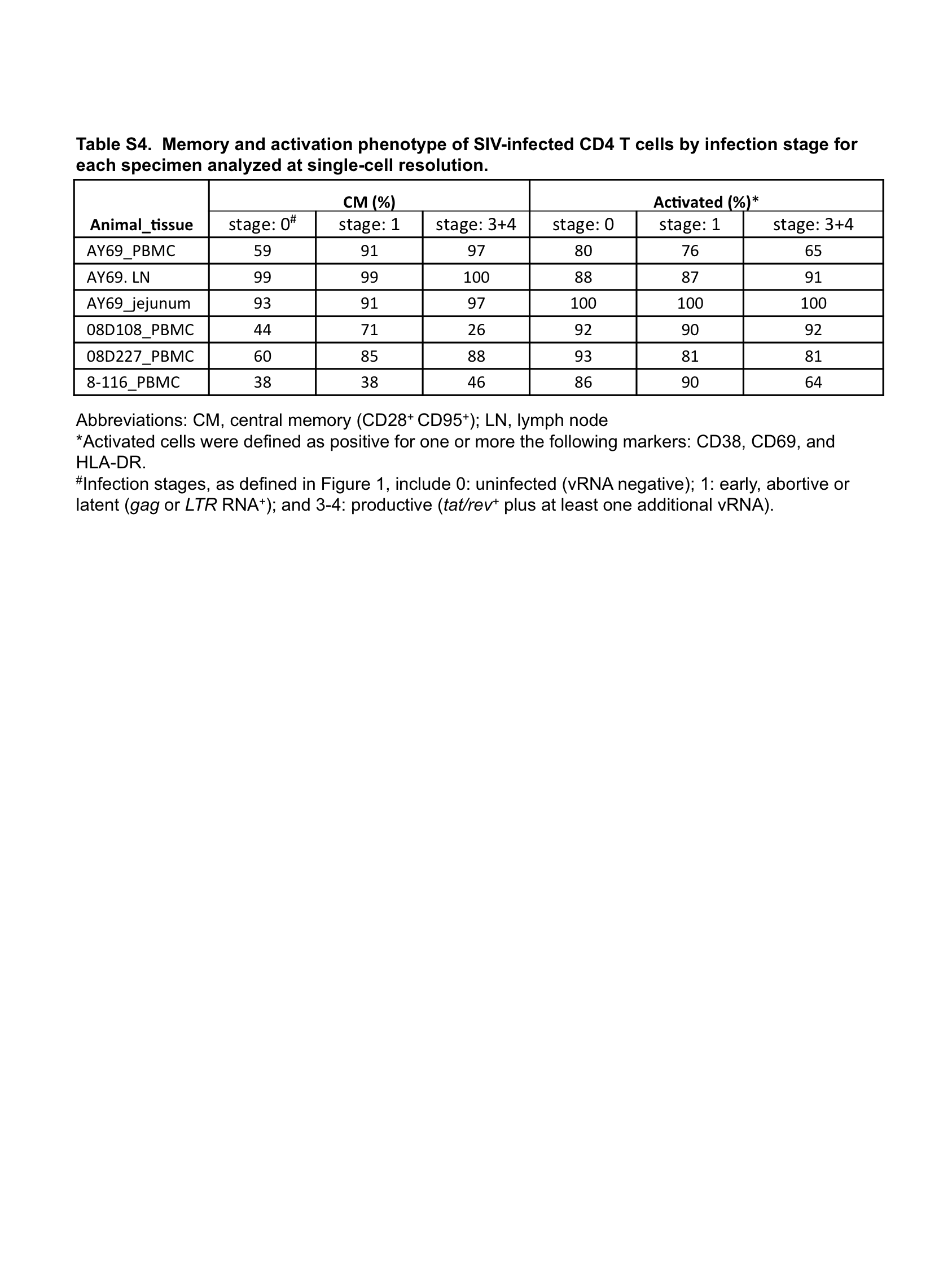

Supplement: S4 Table — (TIF) [file ppat.1006445.s011.tif]
